# Melatonina revierte el daño oxidativo en glándula submandibular de ratas tratadas con Ciclofosfamida

**DOI:** 10.31053/1853.0605.v80.n4.40930

**Published:** 2023-12-26

**Authors:** Fernando Martin Wietz, Evelin Bachmeier, Daniela Josefina Porta, Lorena Moine, Claudio Gastón Dubersarsky, Catalina Melchora Francia, Maria Elena Samar, Maria Angelica Rivoira, Marcelo Adrian Mazzeo

**Affiliations:** 1 Universidad Nacional de Córdoba. Facultad de Odontología. Cátedra de Fisiología. Argentina Argentina; 2 Universidad Nacional de Córdoba. Facultad de Odontología. Cátedra de Fisiología y Cátedra de Estomatología “A” Argentina; 3 Universidad Nacional de Córdoba. Facultad de Odontología. Cátedra de Fisiología. Consejo Nacional de Investigaciones Científicas y Técnicas Instituto de Investigaciones en Ciencias de la Salud. Universidad Nacional de Córdoba. Facultad de Ciencias Médicas; Argentina Argentina

**Keywords:** melatonina, ciclofosfamida, glándula submandibular, ratas wistar, Melatonin, cyclophosphamide, submandibular gland, wistar rats, melatonina, ciclofosfamida, glândula submandibular, ratos wistar

## Abstract

**Objetivo:**

Ciclofosfamida (Cf) produce daño oxidativo en glándula submandibular (GSM) de ratas. En el presente trabajo se evaluó el efecto protector antioxidante de melatonina (MLT) en GSM de ratas tratadas con Cf.

**Método:**

Se utilizaron 40 ratas Wistar machos adultas divididas en 5 grupos (G): G1: control; G2: Control+Etanol: tratados con etanol al 1% durante 10 días consecutivos. Los días 11 y 12 recibieron una dosis de solución salina; G3: Cf: tratados con etanol al 1% durante 12 días, días 11 y 12 recibieron una dosis intraperitoneal (i.p.) de Cf de 50 mg/Kg de pc; G4: Cf + MLT: se administró diariamente MLT (5 mg/Kg pc, intraperitoneal, disuelta en etanol al 1%), días 11 y 12 recibieron Cf igual que G3; G5: MLT: tratamiento 12 días consecutivos con MLT (igual dosis de G4). Los animales fueron anestesiados, extirpándose ambas GSM y sacrificados, previo ayuno 12 hs. Se midió la concentración de ácido úrico (AU), peróxidos lipídicos (PL) y acuosos (PA) y actividad de superóxido dismutasa (SOD) en homogenato de GSM. Análisis estadístico: ANOVA y test de bonferroni, considerando significativo p<0,05.

**Resultados:**

El tratamiento con Cf disminuyó la concentración de AU y la actividad de SOD (AU, mg/mg prot., G1: 2,50±0,68; G2: 2,18±0,13; G3: 0,54±0,09^*^G4: 1,95±0,24^#^, G5: 2,64±0,47,^*^p< 0,01 G3 vs G1, G2, G4; ^#^p< 0,01 G4 vs G3 y G5; SOD, U/mg prot., G1: 4,57±0.95, G2: 4,79±0,94, G3: 2,18±0,53^*^, G4: 5,13±1,10, G5: 5,09±0,39, ^*^p< 0,01 G3 vs G1, G2, G4 y G5). El tratamiento con MLT previno esos efectos. Además, Cf aumentó la formación PL y PA.

**Conclusión:**

MLT mejoró el estado redox en GSM de ratas tratadas con Cf. MLT podría prevenir los procesos oxidativos en GSM producidos por Cf.

## Abstract (en)

**Objetive:**

Cyclophosphamide (Cf) produces oxidative damage in rat submandibular gland (GSM). In the present work we evaluated the antioxidant protective effect of melatonin (MLT) in GSM of rats treated with Cf.

**Methods:**

40 adult male Wistar rats were divided into 5 groups (G): G1: control; G2: Control+Ethanol: treated with 1% ethanol for 10 consecutive days. On days 11 and 12 they received a dose of saline; G3: Cf: treated with 1% ethanol for 12 days, days 11 and 12 they received an intraperitoneal (i.p.) dose of Cf 50 mg/Kg/kg of saline. ) of Cf 50 mg/kg bw; G4: Cf + MLT: MLT (5 mg/kg bw, intraperitoneal, dissolved in 1% ethanol) was administered daily, days 11 and 12 received Cf same as G3; G5: MLT: treated 12 consecutive days with MLT (same dose as G4). After 12 hours of fasting, animals were anesthetized to obtain both submandibular glands, then they were sacrificed. Uric acid (UA), lipid peroxides (LPs), aqueous peroxides (APs) and superoxide dismutase (SOD) activity were measured in submandibular gland homogenate. Statistical analysis: we used ANOVA and Bonferroni test *pos hoc*, considering significant p<0.05.

**Results:**

Cf treatment decreased AU concentration and SOD activity (AU, mg/mg prot., G1: 2.50±0.68; G2: 2.18±0.13; G3: 0.54±0.09* G4: 1.95±0.24#, G5: 2.64±0.47, *p<0.01 G3 vs G1, G2, G4; #p<0.01 G4 vs G3 and G5; SOD, U/mg prot, G1: 4.57±0.95, G2: 4.79±0.94, G3: 2.18±0.53*, G4: 5.13±1.10, G5: 5.09±0.39, *p< 0.01 G3 vs G1, G2, G4 and G5). MLT treatment prevented these effects. In addition, Cf increased PL and PA formation.

**Conclusion:**

MLT improved the redox status in GSM of Cf-treated rats. MLT could prevent oxidative processes in GSM produced by Cf.

## Resumo

**Objetivo:**

Ciclofosfamida (Cf) produz danos oxidativos na glândula submandibular de rato (GSM). No presente trabalho avaliamos o efeito protector antioxidante da melatonina (MLT) no GSM de ratos tratados com Cf.

**Métodos:**

40 ratos Wistar machos adultos foram divididos em 5 grupos (G): G1: controlo; G2: controlo+Etanol: tratado com 1% de etanol durante 10 dias consecutivos. Nos dias 11 e 12 receberam uma dose de soro; G3: Cf: tratado com 1% de etanol durante 12 dias, dias 11 e 12 receberam uma dose intraperitoneal (i.p.) de Cf 50 mg/Kg/kg de soro fisiológico de Cf 50 mg/kg pb; G4: Cf + MLT: MLT (5 mg/kg pb, intraperitoneal, dissolvido em 1% de etanol) foi administrado diariamente, os dias 11 e 12 receberam Cf igual a G3; G5: MLT: tratado 12 dias consecutivos com MLT (mesma dose que G4). Os animais foram anestesiados, ambos os GSM foram removidos e sacrificados, após jejum de 12 h. A actividade de ácido úrico (UA), peróxidos lipídicos (LPs) e peróxidos aquosos (APs) e superóxido dismutase (SOD) foram medidos na homogeneização do GSM. Análise estatística: teste de ANOVA e Bonferroni, considerando p<0,05 significativo.

**Resultados:**

O tratamento Cf diminuiu a concentração AU e a actividade de SOD (AU, mg/mg prot., G1: 2,50±0,68; G2: 2,18±0,13; G3: 0,54±0,09* G4: 1,95±0,24#, G5: 2,64±0,47, *p<0,01 G3 vs G1, G2, G4; #p<0,01 G4 vs G3 e G5; SOD, U/mg prot, G1: 4,57±0,95, G2: 4,79±0,94, G3: 2,18±0,53*, G4: 5,13±1,10, G5: 5,09±0,39,*p< 0,01 G3 vs G1, G2, G4 e G5). O tratamento MLT impediu estes efeitos. Além disso, Cf aumentou a formação de PL e PA.

**Conclusão:**

O MLT melhorou o estado redox no GSM de ratos tratados com Cf. O MLT poderia prevenir processos oxidativos no GSM produzido por Cf.

**CONCEPTOS CLAVE**

**Que se sabe sobre el tema.**

En la actualidad varios reportes clínicos informaron numerosas complicaciones en la cavidad bucal por efecto de la quimioterapia en esquemas oncológicos que utilizan la droga ciclofosfamida. Además de mucositis, inflamación, sangrado de encías y dolor, la hiposialia es comúnmente descripta en estudios observacionales y señalada no solo por los reportes de los pacientes durante el tratamiento con este citostático, sino, además comprobada mediante la técnica de sialometria. También es sabido el carácter no selectivo de esta droga, que puede alcanzar diversas estructuras con alto grado de funcionalidad y mitosis como son las glándulas salivales. Esto representa un estado de toxicidad local que altera de manera significativa la calidad de vida del paciente oncológico, puesto que se ven afectadas funciones tales como la salivación, la fonación, la masticación y la deglución. A partir de estas complicaciones, ocurre un incremento de gastos intrahospitalarios, necesidad de alimentación parenteral y un significativo retraso sobre el tratamiento de base. Por su parte, son escasos los reportes experimentales que analizan desde una perspectiva centrada en la actividad acinar de las glándulas salivales la comprensión de algunas de las manifestaciones clínicas que ocurren en el sistema estomatognático durante los tratamientos farmacológicos que utilizan ciclofosfamida.

**Que aporta este trabajo.**

En tal sentido, el presente trabajo permite comprender a nivel experimental cuales son los cambios oxidativos que suceden a nivel de las glándulas submandibulares, consideradas estas como las principales glándulas salivales encargadas de mantener varios de los parámetros de la homeostasis del sistema estomatognatico. A partir de un modelo animal en el que pueden verificarse numerosas funciones orgánicas extrapolables a los humanos, sería posible corroborar a nivel glandular el nivel de eficacia de algunas barreras antioxidantes como así también, el estrés oxidativo provocado por la toxicidad de esta droga sobre dichas estructuras, lo cual facilitaría la comprensión de algunas de las alteraciones funcionales anteriormente descriptas.

**Divulgación**

La droga Ciclofosfamida (Cf), es muy utilizada para tratar algunos tipos de cáncer. Durante la terapia con este agente farmacológico pueden suscitarse complicaciones en la boca de los pacientes, de manera especial sobre el funcionamiento de las glándulas salivales, situación que altera aún más la calidad de vida del paciente oncológico. Dado que los estudios clínicos no permiten realizar estudios en las glándulas salivales, sino sobre la saliva, el uso de un modelo animal en ratas, facilita la comprensión de algunos mecanismos funcionales sobre estas glándulas que son alterados por esta droga.

## Introducción

Ciclofosfamida (Cf) es una droga ampliamente utilizada asociada a otros citostáticos oncológicos para el tratamiento de tumores sólidos o en esquemas de acondicionamiento para trasplante de médula ósea. Es un agente anticancerígeno alquilante con capacidad de agregar grupos alquilo a grupos electronegativos ^1^ . Es un antineoplásico de la clase de las mostazas nitrogenadas, que debe ser metabolizado en el hígado a su forma activa, la mostaza fosforamida, para ejercer su acción. La naturaleza electrofílica del grupo alquilo permite que el fármaco reaccione con fracciones nucleofílicas de ADN o proteínas, y esto conduce a la transferencia covalente de un grupo alquilo. Además, estos fármacos añaden grupos metilo o alquilo a moléculas a las que no pertenecen, lo que a su vez inhibe su correcta utilización por emparejamiento de bases y provoca una codificación incorrecta del ADN y conduce a mutaciones. Los agentes alquilantes no son específicos del ciclo celular. Cf también posee elevado poder inmunosupresor ^2^ .

Algunos autores reportaron su toxicidad a nivel clínico y experimental en distintos sistemas orgánicos con alto grado de mitosis y actividad funcional como la cavidad bucal ^3^ .

Estudios previos de nuestro laboratorio mostraron que Cf afecta el metabolismo de los hidratos de carbono y la secreción de alfa amilasa salival, disminuyendo la utilización de glucógeno como sustrato metabólico de las glándulas salivales y la función digestiva para sustratos como el almidón. Otro hallazgo interesante de nuestro equipo permitió identificar a nivel experimental el efecto pro oxidativo de esta droga sobre tales glándulas. Estos parámetros fueron utilizados como indicadores de la alteración funcional de las glándulas submandibulares ^4, 5^ .

A su vez, el estudio de algunos sustratos antioxidantes con cierto efecto catalítico como la Superóxido Dismutasa (SOD) y el Ácido Úrico (AU) son responsables del 70% del potencial antioxidante de la saliva. La presencia de cada uno de estos antioxidantes en el fluido salival está condicionada principalmente por la producción glandular, especialmente la de la glándula parótida como en el caso de la SOD. La comprobación realizada por otros autores reportando que la saliva modifica su perfil enzimático en función de las necesidades reductoras del humor en circunstancias de estrés oxidativo, modificó la idea entonces imperante de que se trata de un líquido de composición estable.

Ha sido probado que las respuestas inflamatorias aportan una enorme cantidad de radicales libres, especialmente el anión superóxido y H_2_O_2_. La producción enzimática salival de antioxidantes es eminentemente de origen glandular, siendo susceptible de aumentar su concentración en diferentes tipos de trastornos inflamatorios con efectos sobre la cavidad bucal. La identificación de antioxidantes y el perfil oxidativo medido por peróxidos lipídicos (PL) y peróxidos acuosos (PA) en tejidos, sangre y saliva proporciona una idea sobre la efectividad de los sistemas oxidativos locales o sistémicos ^6^

Por su parte, ha sido probado que la N-acetil-5-metoxitriptamina o melatonina (MLT), es una hormona sintetizada y secretada principalmente por la glándula pineal que además se produce en varios órganos, con importante poder antioxidante. Un claro ejemplo de ello, ocurre a nivel de las glándulas salivales, las cuales la utilizan localmente en favor de su propio metabolismo celular ^7^ .

A partir de estos conceptos hemos hipotetizado que la administración de MLT como coadyuvante farmacológico, podría minimizar el estrés oxidativo sobre sistemas orgánicos con alta capacidad funcional y de mitosis como por ejemplo el sistema estomatognático y en modo particular sobre la función homeostática de las glándulas salivales ^8^ .

En consecuencia, resultaría importante no solo identificar el estrés oxidativo provocado por los fármacos oncológicos que lo producen y la barrera antioxidante que participa en tales procesos, sino también determinar aquellos agentes antioxidantes con capacidad protectora capaces de prevenir o minimizar las complicaciones bucales por acción de los citostáticos.

En base a estos antecedentes en el presente trabajo se evaluó el estrés oxidativo en glándula submandibular de ratas tratadas con ciclofosfamida, analizando el efecto antioxidante de MLT administrada por vía exógena en animales bajo efecto de esta droga antineoplásica.

## Materiales y Métodos

Se utilizaron 40 ratas Wistar machos adultas de 300/350 g de peso corporal (pc) y divididas en 5 grupos con 8 ejemplares cada uno de la siguiente forma: G1: control sin Cf; G2: control+etanol (solución salina 99%+etanol 1%) sin Cf: tratados durante 10 días consecutivos, exceptuando días 11 y 12 que recibieron únicamente una dosis de solución salina sin etanol; G3: Cf: solución salina 99%+etanol 1% durante 12 días. Los días 11 y 12 recibieron además una dosis intraperitoneal (i.p.) de Cf (50 mg/Kg de pc); G4: Cf + MLT: se administró diariamente MLT (5 mg/Kg pc, intraperitoneal, disuelta en solución salina 99%+etanol 1% y los días 11 y 12 recibieron Cf igual que G3; G5: MLT: tratamiento durante 12 días consecutivos con MLT (5 mg/Kg de pc). Los animales previo ayuno 12 horas, fueron anestesiados con una inyección conjunta de ketamina y xylazina de 80 y 12,8 mg/Kg de peso corporal respectivamente, extirpándose ambas GSM y sacrificados mediante maniobra de dislocación cervical. Una vez obtenidas las glándulas submandibulares fueron efectuadas las siguientes mediciones:

### Análisis de las muestras:

#### Concentración de niveles ácido úrico (AU):

Para la determinación de la concentración de ácido úrico (AU) en homogenato de glándula submandibular se utilizó el método enzimático UOD/PAP espectrofotométrico, Trinder Color. Wiener Lab.

#### Determinación de la actividad de superóxido dismutasa (SOD):

Se homogeneizó la GSM con buffer de extracción (fosfato de potasio 50 mmol/L más EDTA 1 MMOL/l, Ph 7,5). Se centrifugó a 1400 rpm a 4°C por 30 min. En el sobrenadante se determinó la actividad en EDTA 1 µM, buffer fosfato de potasio 50 mM pH 7,8, metionina 13 mM, NBT 75 µM y riboflavina 40 µM. Los resultados se expresaron en U SODm/mg de proteína. Las proteínas se midieron por la técnica de Bradford ^9^ .

#### Determinación de peróxidos lipídicos (PL) y acuosos (PA):

Estos compuestos se determinaron en homogenatos de GSM por su capacidad de reaccionar con naranja de xilenol. El procedimiento consistió en incubar a temperatura ambiente y por 30 minutos la muestra con una solución cromógena (10:100 v/v). Para PL, el sulfato amonio ferroso fue reconstituido con hidroxitolueno butilado 4 mM y naranja de xilenol 125 μM en metanol al 90% (Sigma-Aldrich Co., Estados Unidos) Para PA, dicha solución consistió en sulfato amonio ferroso 25 mM en ácido sulfúrico 2,5 M reconstituido con sorbitol 100 mM y naranja de xilenol 125 μM. Ambos fueron medidos a 540 nm y calculados como porcentajes y estandarizar por el contenido proteico ^10^ .

### Análisis estadístico:

Para el análisis estadístico de los resultados fueron utilizados el test de ANOVA y test *pos hoc* de Bonferroni, considerando significativo un p valor <0,05.

El presente proyecto fue aprobado por el Comité para el Cuidado y Uso de Animales de Laboratorio (CICUAL) según Resolución 89/19 dependiente de la Facultades de Ciencias Médicas y Odontología de la Universidad Nacional de Cordoba, siguiendo además las normas internacionales NIH para el manejo de animales de experimentación.

## Resultados

La concentración de AU fue más baja en G3 respecto a todos los grupos (*p< 0,001), además la concentración de AU en G4 fue significativamente menor en G4 con respecto a G5 (*p< 0,001) (Figura 1). Por otro lado, la actividad de la SOD en G3 fue más baja con respecto a los demás grupos de tratamiento. (Figura 2). En la evaluación de la expresión de prooxidantes, la concentración tanto de peróxidos lipídicos (PL) como de peróxidos acuosos (PA) fue superior en G3 con respecto a todos los grupos de tratamiento. (Figuras 3 y 4). Podemos concluir en este sentido que el tratamiento con Cf disminuyó los niveles de AU ý la actividad de SOD en GSM. Mientras que MLT revirtió estos parámetros. Además, Cf aumentó la producción de PL y PA, mientras que los animales tratados con MLT mejoraron estos parámetros. Por lo tanto, MLT atenuó el efecto oxidativo producido por Cf.

**FiguraN°1:**
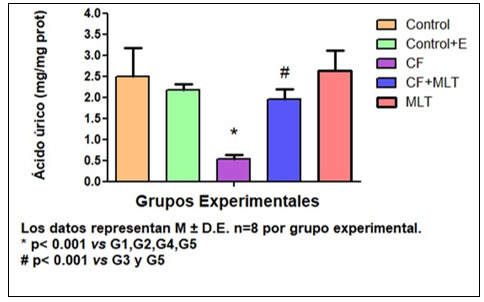
Evaluación de la concentración de ácido úrico (AU) en GSM. Los datos representan M ± D.E n=8 por grupo experimental.

**Figura N° 2:**
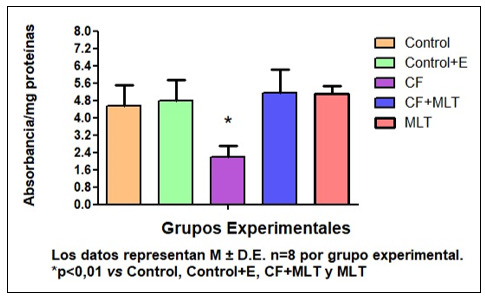
Actividad de Superóxido dismutasa (SOD). Se evaluó la actividad de SOD en homogenato de GSM como se describe en la sección de metodología. Los datos representan M ± D.E n=8 por grupo experimental.

**Figura N° 3:**
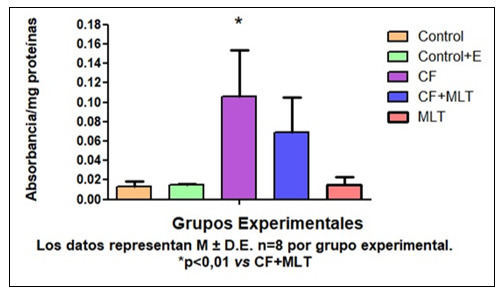
Expresión de peróxidos lipídicos en homogenatos de GSM en los distintos grupos experimentales. Los datos representan M ± D.E n=8 por grupo experimental.

**Figura N° 4:**
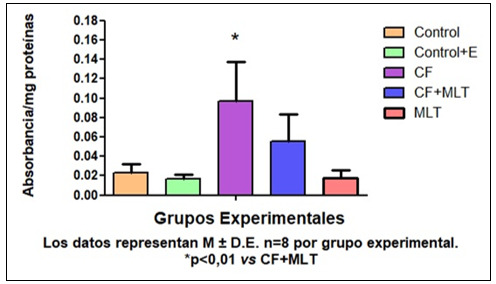
Expresión de peróxidos acuosos en homogenatos de GSM en los distintos grupos experimentales. Los datos representan M ± D.E n=8 por grupo experimental.

## Discusión

El oxígeno es esencial para la vida de los organismos aeróbicos. Sin embargo, bajo determinadas circunstancias, las formas reactivas de oxígeno o radicales libres pueden ser letales para los tejidos de aquellos seres vivos que dependen de ésta molécula para vivir ^11^ .

En condiciones fisiológicas, existe un equilibrio por acción del sistema antioxidante endógeno frente a diversas sustancias oxidantes. El estrés oxidativo ocurre cuando el equilibrio entre la formación y eliminación de especies reactivas de oxígeno (ROS) y las especies reactivas de Nitrógeno (NOS) se alteran en beneficio por un exceso de los mismos ^12^ .

ROS y NOS participan como causa o consecuencia en diversas disfunciones orgánicas. Numerosas patologías están directamente relacionadas con estados hiperoxidativos. Entre las más importantes encontramos anemias, enfermedades neurodegenerativas, enfermedad pulmonar, patología renal, enfermedades autoinmunes, fibromialgia y cáncer ^13^ .

Por otra parte, ha sido demostrado que diversos fármacos tienen capacidad potencial para generar estrés oxidativo dentro de los que se destacan los agentes antineoplásicos ^14^ .

Tal como fuera reportado por otros autores y nuestro equipo, la administración de citostáticos afecta además de las células tumorales, aquellos órganos y tejidos con alta tasa de actividad funcional y mitosis como la cavidad bucal, siendo las glándulas salivales una de las estructuras blanco más comúnmente involucradas. Esto altera la homeostasis del sistema estomatognático, provocando una reducción de la calidad de vida de los pacientes sometidos a tratamiento oncológico específico, que se evidencia en la imposibilidad de llevar a cabo funciones como la formación del bolo alimenticio, digestión parcial del almidón, perdida de la capacidad defensiva local, la deglución, alteración de la percepción de las sustancias sápidas, inflamación gingival y de tejidos blandos, infecciones oportunistas, incremento de caries dentales, fonación y dolor ^15, 16^ .

Recientemente, nuestro laboratorio reportó en pacientes con indicación de trasplante de médula ósea un incremento significativo tanto de ácido úrico y de la enzima superóxido dismutasa salival, durante la terapia de acondicionamiento ^17^ .

A nivel experimental, informamos cambios histológicos, funcionales y oxidativos en GSM de ratas tratadas por acción de Cf, comúnmente utilizada en tumores sólidos y en terapias de acondicionamiento para trasplantes de medula ósea ^18^ .

Con el propósito de minimizar los efectos del estrés oxidativo sobre distintas estructuras orgánicas por efecto de la quimioterapia, en consonancia con otros autores fueron utilizados algunos agentes antioxidantes como la vitamina C y E tanto en ensayos experimentales como en estudios clínicos con resultados controversiales ^19, 20^ .

Recientes estudios reportaron que la MLT, tiene un potente efecto antioxidante. Debido a un elevado potencial redox, esta sustancia cede electrones muy fácilmente haciendo que actúe como un potente agente reductor. La actividad antioxidante de la MLT estaría dada por su capacidad recolectora de radicales libres de las que dispondría un organismo contra diversas injurias exógenas, incluso las farmacológicas ^21, 22^ .

Sus efectos parecieran ser superiores a las vitaminas E y C descriptas anteriormente, por ser una molécula tanto liposoluble como hidrosoluble permitiéndole difundir con gran facilidad tanto en la membrana, como en el núcleo o en la mitocondria celular. Además, tiene la función de depurar especies reactivas de oxigeno o nitrógeno ^23^ .

Es ampliamente conocido que, además de la glándula pineal, otros órganos sintetizan MLT tales como la retina, células del sistema inmune, intestino, médula ósea, ovario, testículo, cerebro, hígado y corazón, entre otros. No obstante, a diferencia de la que se produce a nivel pineal, la melatonina sintetizada en estos órganos no sale a la circulación, sino que es utilizada con fines protectores a favor del órgano o tejido que la produce ^24, 25^ .

Varios autores postularon la presencia de MLT en las glándulas salivales mayores, tanto en muestras biológicas obtenidas de humanos como en ratas. Anteriormente se creía que la melatonina pasaba por difusión pasiva del plasma a las glándulas salivales. Actualmente se ha identificado por inmunohistoquímica que las mismas glándulas salivales - al igual que otros tejidos y órganos- tienen potencial para sintetizar y secretar su propia melatonina ^26^ .

Con el fin de determinar los diferentes efectos farmacológicos de MLT sobre diversos sistemas orgánicos, se ha probado un amplio rango de concentraciones a nivel experimental. Por ejemplo, en ratas las dosis ensayadas fueron entre 3 y 200 mg/kg de peso corporal, sin efectos tóxicos de relevancia. En tanto que en humanos fueron ensayadas dosis entre 1mg hasta 1 gramo de melatonina durante 30 y 90 días, sin efectos adversos probados ^27, 28^ .

Considerando los reportes de otros trabajos, desarrollamos este ensayo experimental administrando MLT por vía sistémica con el objeto de minimizar los efectos deletéreos de Cf sobre las glándulas salivales. Desde esta perspectiva y en coincidencia con otros investigadores, la administración de MLT como coadyuvante farmacológico aplicada con fines terapéuticos sobre sistemas orgánicos con alta capacidad funcional, tal como ocurre con las glándulas salivales, favorecería una disminución del estrés oxidativo ^29^ .

A partir de nuestros hallazgos, resulta importante no solo haber identificado el daño oxidativo provocado por Cf, sino también haber reconocido la capacidad protectora de la MLT sobre estas glándulas por acción deletérea de este citostático. En tal sentido, podemos inferir que la administración exógena de MLT en la dosis ensayada, permitió revertir y equilibrar el estado redox de las GSM, tal como fue demostrado por una normalización de la batería antioxidante junto a una reducción significativa del estrés oxidativo. En la literatura consultada no hemos identificado otros trabajos con los cuales contrastar nuestros resultados. Si bien las glándulas salivales en condiciones fisiológicas tendrían capacidad para utilizan localmente MLT en favor de su propio metabolismo celular, podríamos hipotetizar que frente a la administración de ciclofosfamida dicha producción resultaría insuficiente. En tal sentido la administración suplementaria de MLT actuaría como agente coadyuvante antioxidante.

En síntesis, nuestro estudio permitiría dar continuidad a futuras investigaciones tomando como punto de partida que la administración exógena de MLT, sumada a la batería antioxidante producida por el propio organismo, tendrían capacidad para minimizar los efectos pro oxidativos ocasionados por la administración de Cf.

Resulta interesante seguir realizando nuevos estudios acerca del comportamiento de la MLT como "antioxidante multipropósito", y su efecto sobre el estrés oxidativo en las glándulas salivales ocasionados por acción de este citostático y otras posibles drogas oncológicas que, por su carácter no selectivo, pueden alcanzar otras estructuras funcionales de la cavidad bucal ^30^ .
